# Development and tuning of an original search engine for patent libraries in medicinal chemistry

**DOI:** 10.1186/1471-2105-15-S1-S15

**Published:** 2014-01-10

**Authors:** Emilie Pasche, Julien Gobeill, Olivier Kreim, Fatma Oezdemir-Zaech, Therese Vachon, Christian Lovis, Patrick Ruch

**Affiliations:** 1 Division of Medical Information Sciences (SIMED), University Hospitals of Geneva and University of Geneva, Rue Gabrielle-Perret-Gentil 4, 1211 Geneva 14, Switzerland; 2 Bibliomics and Text-Mining Group (BiTeM), Information Science Department, University of Applied Sciences, Route de la Drize 7, 1227 Carouge, Switzerland; 3 Swiss Institute of Bioinformatics (SIB), Rue Michel Servet 1, 1211 Geneva 4, Switzerland; 4 Novartis Institute for BioMedical Research - Text Mining Services (NIBR-IT/TMS), Novartis Pharma AG, Postfach, 4002 Basel, Switzerland

## Abstract

**Background:**

The large increase in the size of patent collections has led to the need of efficient search strategies. But the development of advanced text-mining applications dedicated to patents of the biomedical field remains rare, in particular to address the needs of the pharmaceutical & biotech industry, which intensively uses patent libraries for competitive intelligence and drug development.

**Methods:**

We describe here the development of an advanced retrieval engine to search information in patent collections in the field of medicinal chemistry. We investigate and combine different strategies and evaluate their respective impact on the performance of the search engine applied to various search tasks, which covers the putatively most frequent search behaviours of intellectual property officers in medical chemistry: 1) a *prior art search *task; 2) a *technical survey *task; and 3) a variant of the *technical survey *task, sometimes called *known-item search *task, where a single patent is targeted.

**Results:**

The optimal tuning of our engine resulted in a top-precision of 6.76% for the *prior art search *task, 23.28% for the *technical survey *task and 46.02% for the variant of the *technical survey *task. We observed that co-citation boosting was an appropriate strategy to improve *prior art search *tasks, while IPC classification of queries was improving retrieval effectiveness for *technical survey *tasks. Surprisingly, the use of the full body of the patent was always detrimental for search effectiveness. It was also observed that normalizing biomedical entities using curated dictionaries had simply no impact on the search tasks we evaluate. The search engine was finally implemented as a web-application within Novartis Pharma. The application is briefly described in the report.

**Conclusions:**

We have presented the development of a search engine dedicated to patent search, based on state of the art methods applied to patent corpora. We have shown that a proper tuning of the system to adapt to the various search tasks clearly increases the effectiveness of the system. We conclude that different search tasks demand different information retrieval engines' settings in order to yield optimal end-user retrieval.

## Background

Over the last decades, the size of patent collections has strongly increased. In 2009, it was estimated that there are globally about 50 million patents [1] with about 15-20 million related to medicinal chemistry and life sciences, which represents a corpus of knowledge comparable to the content of MEDLINE with the noticeable difference that MEDLINE contains only relatively short abstracts. These collections constitute an important and high-quality source of knowledge. However, while search engines to navigate the post-omics biomedical literature [2] have benefited from the availability of excellent text mining services and implement recent retrieval techniques [3], most commercial and freely available tools to access documents stored in patent libraries use rather traditional - yet often appreciated - search models (e.g. Boolean-like search engines, no related search skills, etc.).

Recently, several evaluation campaigns [4,5] have attracted attention on the importance of the role of information retrieval in the field of the intellectual property. Prestigious universities, but also corporate research centres, have regularly participated in such evaluation campaigns; thus fostering the development of specialized patent retrieval engines to perform patent-related competitive intelligence tasks. One of the most popular competitions to evaluate and compare search engines, the Text REtrieval Conferences (TREC) [6], has lately set up an information retrieval track dedicated to patent search for chemistry, called TREC-Chem [7]. To evaluate search engines, two tasks have been defined: a *prior art *(PA) task and a *technical survey *(TS) task. The PA task aims to determine how systems can help recovering the prior art of a given patent. For this task, queries are full-text patents. The TS task is a traditional *ad hoc *retrieval problem. With relatively short queries (i.e. typically a few sentences), the systems must retrieve a set of relevant patents that fulfil a particular information need. In this context, a collection of about 1.3 million patents is provided to participants, as well as queries for both tasks. Relevance judgments are defined after submission of the runs. The participants of such competitions have explored various strategies.

Some groups have investigated the use of ontologies to improve information retrieval effectiveness. In particular, query expansion based on chemical terms has been tested [8-11], which resulted in a moderate improvement of the performance. More convincing, Jimeno-Yepes *et al. *[12] reported that normalization was useful to search the medical literature provided that the onto-terminological resources (e.g. synonyms) are carefully curated. Similarly, a study conducted by Ruch *et al. *[13] reported that the effectiveness of normalization and query expansion was depending on the entity type. Indeed, it was showed that normalization and expansion of genes and gene products degraded the precision of search in MEDLINE during the TREC Genomics [14] competition, while normalizing chemical, pathological, organism-related and anatomical concepts was moderately effective. We also acknowledge that more advanced string normalization algorithms (e.g. [15] or [16]) could have improved the effectiveness of the normalization components but the impact on the efficiency remains unknown.

For *prior art *tasks, queries are usually full-text patents, resulting in very long queries. However, investigations have shown that some features of a patent may have a negative impact on the information retrieval performance and therefore feature selection should be performed at indexing or retrieval time. Cetintas *et al. *[8] has proposed an approach where full title and selected words from the abstract, claims and description are used to construct the query. Mejova *et al. *[17] investigated a different approach by focusing on the data contained in the claims, but results were relatively disappointing.

Several groups have explored the use of citations, assuming that highly relevant patents are more likely to be cited more often than less relevant patents [18]. The approach, pioneered by Gobeill *et al. *[9], which was ranked #1 during the TREC evaluation campaign, performed a citation-based re-ranking of the initial results, while, Gurulingappa *et al. *[11] proposed to return a ranked list of the patents cited in the initial results. Both approaches had a highly significant positive impact on the performance of the search engines.

Some authors [19] reported that using International Patent Classification (IPC) codes with four values allowed retrieving the totality of the state of the art. In the same vein, Criscuolo and Verspagen [20] affirmed that 65% to 72% of the state of the art was collected using IPC codes. On the opposite, previous experiments conducted for TREC-Chem by several of the competitors, e.g. [11,21] tend to show that IPC classes were not of great interest for *prior art search*.

Although search by chemical structure is important in the domain, only few TREC competitors have explored this aspect [22,23]. Structure search is indeed central in commercial patent retrieval systems, although it is worth observing that about two thirds of the patents related to the chemical domain do not contain structural information.

Based on the experience we have acquired during TREC competitions [9,21,24], our objective here is to report on the development and tuning of an original search engine for patent collections related to the pharmaceutical domain, which includes various fields of health and life sciences including biochemistry, biotechnology and medical technology. Moreover, in contrast to previous works done during the TREC competitions, the objective here is to develop an operational search engine in the working environment of a large pharmaceutical company. This means that the tested methods must be effective, efficient and scalable enough to adapt to high volumes of data. The finally delivered search engine has been implemented as a web application that we briefly describe at the end of the paper. This paper is an extended version of an original extended abstract submitted to NETTAB 2012 [25].

## Methods

### Data

A collection of more than 13 million patents stored in an Oracle Database Management System provided and maintained by IBM Almaden for Novartis as part of a collaboration project between Novartis/NIBR-IT and IBM has been made available for this project. The resource is supposed to cover all fields of applications relevant for the life and health sciences and its content is judged as highly relevant by Novartis' users. A set of 1'004'868 patents has been randomly selected out of this collection. We decided to work with a sizeable sample and not with the whole collection in order to speed up the prototyping process. Thus, 33 days would have been necessary to only extract the data from the Oracle database. Such sampling is inspired by the TREC patent retrieval campaign, which used a 10% subset of the MAREC collection [7]. The ultimately delivered system uses a Hadoop/HDFS file system that we do not describe in this report. The content of the patents has been extracted using SQL queries and stored in files using an ad hoc XML format.

### Evaluation

The evaluation of the search engine is performed regarding two search tasks: a PA task and a TS task. These two tasks have different objectives and constraints and may therefore require different tuning of the search engine. The reason we decided to focus on these two tasks is that they cover the putatively most frequent search behaviours of intellectual property officers in medicinal chemistry.

Therefore, we created the following benchmarks (i.e. a set of queries and relevance judgments): a benchmark dedicated to the PA task and two benchmarks dedicated to the TS task. We decided to create two benchmarks for the TS task because the initial one, based on the TREC-Chem methodology, was nearly too small to provide statistically significant evaluations.

The first benchmark is used to simulate and evaluate a PA task, also called *related patent search*. We use the same methodology as proposed by TREC-Chem 2009 for a similar PA task [7]. This benchmark is constituted of 96 long *topics *(or queries). Each topic corresponds to the title, abstract and claims of a given patent. For these experiments, the relevance judgments are generated out of the set of patents cited as prior art by the topics. Only cited patents that are represented in the sample collection are used in the relevance judgements because patents - as well as other reports - cited but not found in the collection cannot be retrieved by the engine.

**Figure 1:**
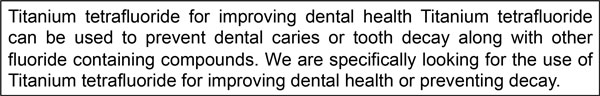
**Example of a topic of the benchmark for the traditional *technical survey *task**.

The second benchmark is used to evaluate the engine in a traditional TS task. In such a task, queries are usually limited to a few keywords. This benchmark contains 24 short topics (Figure 1), corresponding to the TREC-Chem 2010 [26] and 2011 [27] TS topics. Relevance judgments are provided by TREC and have been pre-processed to filter out patents not available in the sample collection we are using.

**Figure 2:**
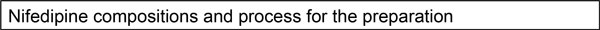
**Example of a topic of the benchmark for the variant of the *technical survey *task**.

The third benchmark is used to evaluate a variant of the TS task, where a single patent is targeted, using a *known-item search *methodology [28]. It is constituted of 514 short topics (Figure 2). Each topic contains ten words randomly selected from the title, abstract or claims of a given patent. In this set of experiments, the relevance judgments for each topic correspond to the patent from which the words are originally extracted. In that setting, there is only one patent considered as relevant for each query.

The tuning of the system is based on the maximization of the top-precision, also called P0 or *mean reciprocal rank*, for the three different tasks. This measure evaluates the precision of the first returned result by the search engine. In our preliminary experiments, we focus on this metrics since it provides a sound estimate of the retrieval effectiveness of the system for the three benchmarks. We also evaluate the mean average precision or precision interpolated over different recall points (MAP, which takes into account up to 1000 results), but such measure cannot be applied to *known-item search *tasks, since a *known-item search *task assumes only one relevant document per query. In any case, we would argue that P0 also provides a better metrics than MAP to assess the final usability of the system as we were told by our Novartis users that they might simply decide to ignore patents, which would not be found in the top 5 or 10 search results.

### Methods

In this section, we present the main aspects of our approach. The building of our search engine is based on four steps: 1) collection pre-processing; 2) collection indexing; 3) documents retrieval and 4) documents ranking.

First, regarding the pre-processing of the patent collection, we evaluate the impact of an ontology-driven normalization of the patent content. Three terminologies are used for these experiments. The Medical Subject Headings (MeSH) are used to normalize entities related to anatomy, chemical substances, devices, disorders, genes, geography, populations, procedures and species. The Gene Ontology (GO) is used to normalize entities describing biological processes, cellular components and molecular functions. Finally, the Caloha terminological resource [29] is used to normalize entities such as cells and tissues. Normalized terms are stored as metadata. Three settings are tested: no normalization is performed; the normalization is performed on the following fields only: title, abstract and claims (referred in the following with the acronym "TAC"); the normalization is performed on all fields, i.e. title, abstract, claims and description (referred in the following with the acronym "TACD").

Second, the collection is indexed using the Terrier Information Retrieval platform, which provides more advanced document ranking models than Lucene-based platforms. Schemas based on the deviation from randomness are indeed not available in Lucene while the Lucene implementation of BM25 is knowingly suboptimal [30]. However, Terrier is currently not able to perform incremental indexing. As a consequence, we need to optimize our approach regarding the indexing time so that the index can be updated within a reasonable timeframe. Indexing is performed using baseline settings, with Porter stemming. Different indexes of the collection are generated to evaluate the impact of our strategies. First, we attempt to evaluate the impact of the description field - a CPU-intensive field both at normalization and indexing time - on the search effectiveness of the engine with the three use cases. Indeed, for sake of efficiency (in particular indexing time), we attempt to select only the most content-bearing sections of the patent. This investigation requires performing two distinct indexes: the first one based on the content of the whole patent and the second one using the patent but the description field. Second, we evaluate the impact of the metadata field, to determine whether our onto-terminological normalization strategies bring useful additional information. For this investigation we created three indexes of the patent collection: the first one includes the whole patent excluding the metadata field (i.e. no normalization is performed); the second one includes the whole patent and the TAC metadata and finally, the third one includes the whole patent and the TACD metadata.

Third, patents are retrieved using the Terrier Information Retrieval platform. We evaluate the impact of the search model. Two search models are tested: the Okapi BM25 [31] and PL2, a model based on Poisson estimation from randomness [32], which outperformed Okapi in very similar contexts [21].

Fourth, we evaluate the use of co-citation networks to improve our results by re-ranking patents. With this approach, we favour patents, which are the most cited ones in the collection disregarding the content of the query. We rank all patents by the number of time each patent is cited by the others; thus building a large co-citation matrix. Then, we combine through linear combination this ranking with the results of the query as originally returned by the retrieval engine.

Additionally, we also attempt to evaluate the impact of the use of IPC classes to refine the queries. Thus, we simply add the IPC codes to the query and execute a new run. For the benchmarks used for the PA task and the variant of the TS task, the IPC codes assigned to the originally filed patents by the patent officer are used, while for the benchmark built for the traditional TS task, the IPC codes were assigned by the evaluators of TREC-Chem.

## Results

### *Prior art search* task

The results of the tuning of the search engine for the PA task are presented in table 1. The initial run reached a P0 of 2.20%. We observed that removing the description field from the index resulted in an increase of the precision at high ranks from 2.20% to 2.87% (+23%, p<0.01). Consistent with this observation, we also noticed that the use of TAC metadata, which was generated based only on the content of the title, abstract and claims improved the precision of our results compared to the TACD metadata, which includes in addition the description, from 2.87% to 3.64% (+26%, p<0.01). No significant difference is observed when using an index without metadata or an index with TAC metadata. Concerning the weighting model, we observed that BM25 performed much better than the deviation from randomness weighting schema we tested, with an increase of the P0 from 2.87% to 5.36% (+87%, p<0.01). Our experiments focused on the feature selection and combination steps; therefore we assume the results reported here are weighting schema-independent; in particular because BM25 can be regarded as a strong baseline, see e.g. [30]. Regarding the use of citation-based network, we can assume that it is an appropriate functionality for PA task with an increase of the P0 from 5.36% to 6.76% (+26%, p<0.01). A query-by-query analysis provided interesting additional information. Twenty-seven topics were better answered when the co-citation network was used; five topics resulted in lower results, while 64 topics remained simply unchanged. For instance, the topic defined by using the title/abstract/claims of the patent *US6007222 *had two relevant patents retrieved by our system: one was ranked #38 and the other one #96. With co-citation boosting, these two patents were pushed up to rank #1 and #2. Thus, the MAP of this specific query increased from 2.31% to 58.33%. Finally, concerning the use of IPC codes, we obtained no improvement when adding them to the query, with a significant decrease of the P0 from 6.76% to 5.88% (-13%, p<0.01).

**Table 1:** Tuning of the search engine regarding the *prior art search *task.

Strategy	Tuning	P0	MAP
Impact of the description field	Description	2.20%	1.34%
	**No description**	**2.87%**	**1.52%**

Impact of the metadata field	**No metadata**	**3.64%**	**1.93%**
	Metadata on TAC	3.63%	1.92%
	Metadata on TACD	2.87%	1.52%

Impact of the weighting model	PL2	2.87%	1.52%
	**BM25**	**5.36%**	**3.47%**

Impact of the co-citation network	Without re-ranking	5.36%	3.47%
	**With re-ranking**	**6.76%**	**4.22%**

Impact of the use of IPC codes	**No IPC codes**	**6.76%**	**4.22%**
	IPC codes	5.88%	3.66%

The best tuning for each strategy is displayed in bold. P0 indicates the top-precision and MAP represents the mean average precision.

### *Technical survey *task

The results of the tuning of the search engine for the traditional TS task are presented in table 2 and for the variant of the TS task in table 3. The initial runs obtained a P0 of 15.87% for the traditional TS task and of 23.63% for the variant of the TS task. Similarly to the PA task, we observed that removing the description field from the index had a positive impact on the results of the TS tasks with an increase of the P0 from 15.87% to 19.51% for the traditional one (+19%, p<0.01) and from 23.63% to 33.59% for the variant of the TS task (+30%, p<0.01). Similar observations had also been made regarding the use of metadata. Indeed, the use of TACD metadata decreased the precision compared to TAC metadata from 30.30% to 19.51% for the traditional TS (-35%, p<0.01) and from 35.02% to 33.59% for the variant TS (-4%, p<0.01). No significant difference was observed when using an index without metadata or an index with the TAC metadata. Regarding the weighting model, we observed that BM25 performed better than the PL2 schema with a slight increase of the P0 from 19.51% to 20.05% for the traditional TS (+3%) and more significantly from 33.59% to 40.86% for the variant TS (+22%, p<0.01). Concerning the use of the co-citation network, the improvement was significant for the traditional TS only with an increase of the P0 from 20.05% to 21.24% (+6%, p<0.01), however the size of the available TREC query set (N = 24) used in this experiment is nearly insufficient to draw a firm conclusion. Finally, regarding the use of IPC codes, a significant improvement of the precision was observed for both benchmarks with an increase of the P0 from 21.24% to 23.28% for the traditional TS (+9%, p<0.01) and from 40.87% to 46.02% for the variant TS (+13%, p<0.01).

**Table 2:** Tuning of the search engine regarding the traditional *technical survey *task.

Strategy	Tuning	P0	MAP
Impact of the description field	Description	15.87%	1.49%
	**No description**	**19.51%**	**1.48%**

Impact of the metadata field	**No metadata**	**30.92%**	**1.74%**
	Metadata on TAC	30.30%	1.60%
	Metadata on TACD	19.51%	1.48%

Impact of the weighting model	PL2	19.51%	1.48%
	**BM25**	**20.05%**	**1.59%**

Impact of the co-citation network	Without re-ranking	20.05%	1.59%
	**With re-ranking**	**21.24%**	**1.60%**

Impact of the use of IPC codes	No IPC codes	21.24%	1.60%
	**IPC codes**	**23.28%**	**1.62%**

Results in bold are the most effective and are therefore selected to perform further experiments. P0 indicates the top-precision and MAP represents the mean average precision.

**Table 3:** Tuning of the search engine regarding the variant of the *technical survey *task.

Strategy	Tuning	P0
Impact of the description field	Description	23.63%
	**No description**	**33.59%**

Impact of the metadata field	No metadata	34.78%
	**Metadata on TAC**	**35.02%**
	Metadata on TACD	33.59%

Impact of the weighting model	PL2	33.59%
	**BM25**	**40.86%**

Impact of the co-citation network	Without re-ranking	40.86%
	**With re-ranking**	**40.87%**

Impact of the use of IPC codes	No IPC codes	40.87%
	**IPC codes**	**46.02%**

The tuning displayed in bold is selected for the further strategies. P0 indicates the top-precision.

### Implementation

**Figure 3:**
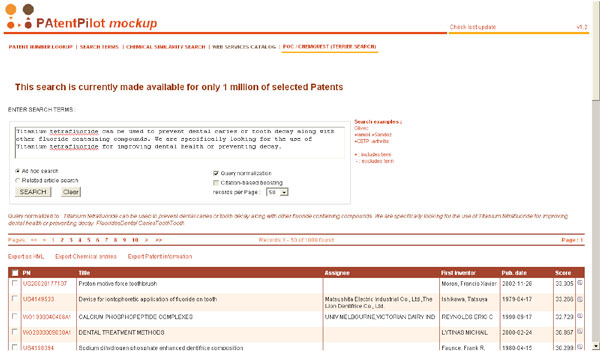
**Welcome page of the Novartis search application**. Example of an *ad hoc search *for the topic mentioned in figure 1.

**Figure 4:**
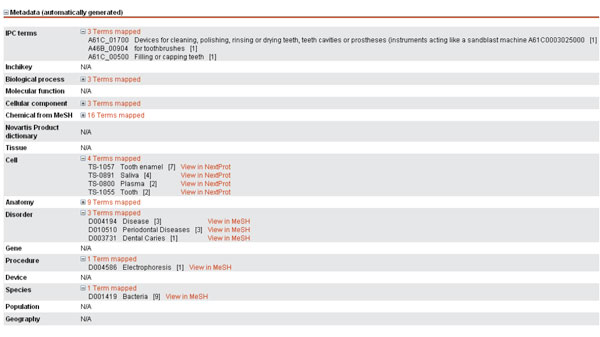
**Example of metadata**. Example of normalized metadata automatically assigned to a patent.

The final search engine has been embedded in the Novartis' engine (so-called PAtentPilot, shown in Figure 3) as a web service. The two search modes described in this paper are proposed: *ad hoc search *(or *technical survey*) and *related patent search *(or *prior art search*). An ontology-driven normalization of the query is performed, if selected by the user, using a richer set of terminologies, including Novartis' proprietary resources. Citation-based boosting is also available as an option. All relevant patents are displayed in a table and can be further explored (Figure 4), displaying among others the metadata automatically assigned to the patent.

## Discussion

### Comparison with other systems

A comparison of the results of the system presented in this paper and other systems, such as those participating in the TREC-Chem competition should be conducted carefully. Indeed, despite the use of a similar methodology, both the collections and the sets of queries are different. However, we can state that our results are consistent with state of the art results reported during TREC experiments. Indeed, regarding the PA task of TREC-Chem 2009 [7], the mean reciprocal rank of participants' systems was ranging from 1.4% to 50%, with a median value of 11%. Our best-tuned system reached a mean reciprocal rank of 6.76%. Such score could be regarded as low, however it is important to notice that the mean reciprocal rank (precision of the first returned patent) can reach 50% meaning that the top-returned patent is cited in the prior-art half of the time. The relatively low mean average precision is probably due to a recall problem: there are many relevant patents, which are simply not cited by the authors. Other experiments [33] performed with patent collections in unrestricted domains show that only the top-10 returned patents are highly relevant for passage retrieval; thus suggesting the precision of retrieval systems in patents decreases sharply after the top ranks.

### Impact of description

The main, as well as most surprising observation of our experiments is that the description field did not improve our results for any of the three benchmarks. We thus decided to remove the description field from the engine's indexes, which resulted in faster indexing (i.e. 45% faster in our experimental settings). Further, the removal of the description field considerably reduced the size of indexes (i.e. one order of magnitude). Furthermore, our observations showed that the description field should also be discarded when generating the normalized version of the patent, which also resulted in a significant gain of time for the normalization process (i.e. 30% faster). This is of great interest for the efficiency of further updates of our system. However, more recent findings suggest that descriptions should not be always discarded. Thus, to perform passage retrieval tasks, it seems that the description field should be parsed taking into account the position of the information in the field: in particular passages occurring at the beginning and end of a section seem strongly associated with relevant passages [33,34].

### Impact of normalization

Similar to state of the art observations, our terminology-driven normalization modules have basically no effect on the search skills of the engine. Normalization of title, abstract and claims did not improve - neither decrease - significantly the search effectiveness of our engine. This observation could be explained by the quality of the normalization itself. Indeed, the normalization of the patent content has been performed automatically. We were not able to directly measure the precision of our normalization process due to the absence of benchmarks for such a large set of biomedical entities (molecular functions, cellular components, cell types, tissues, diseases, chemical compounds, metabolic pathways, species, sign and symptoms, etc.). Another reason could be, as mentioned by Ruch *et al. *[13], that some entity types have a positive impact while others may decrease the results. As a further experiment, it would be interesting to evaluate the impact of the normalization by entity types.

### Impact of the citation network

The impact of the citation network clearly improves the effectiveness of the search for the PA task. In some previous experiments, a +100% improvement has been reported, which is probably an overestimation [21]. A future research direction could be to characterize a priori which queries are likely to benefit from the citation network, because in some cases, the citation network remains detrimental.

### Impact of the IPC codes

Our experiment of using IPC codes to filter the set of results had a positive impact for the TS task. Moreover, a stronger impact could have been reported for the benchmark representing the traditional TS if IPC codes were attributed to each topic as only 6 out of 24 queries were provided with IPC codes. Thus, we can assume that using an interactive IPC classifier [35,36] for *ad hoc search *could have a beneficial effect on the effectiveness of the search engine. In contrast, the length of the input (an extract of the patent) for the PA makes obviously the use of IPC descriptors of less value, which is consistent with the state of the art [11,21].

## Conclusion

We have thus presented the development of a search engine dedicated to patent search, based on the state of the research methods applied to patent corpora. We have shown that a proper tuning of the system clearly increases the effectiveness of the system. We conclude that different search tasks, such as *related patent search *and *ad hoc search*, demand different information retrieval settings in order to yield optimal effectiveness. The "one engine fitting all needs" is obviously suboptimal. Whatever basic search engine platform is selected, the tuning (selection of sections, normalization, weighting schema, use of co-citations, etc.) must be precisely adapted to the user's information requests (e.g. *ad hoc *vs. retrieval by documents); therefore, the search engine is currently being deployed as a complement of existing commercial search solutions and additional studies would be needed to assess the practical usability of the engine. Nevertheless some general scientific statement can be derived from our experiments: we thus recommend using indexes of all fields except the description for all search tasks. Similarly, citation networks should be integrated in the search pipeline for the *related search *task (*prior art search*), while IPC classification seems effective for *ad hoc search *queries (*technical survey*). As reported elsewhere, BM25 weighting schema seems to provide state of the art performances for basic search tasks. Finally, further experiments could be needed to determine whether the fusion of different weighting schema could provide some improvements for patent retrieval like often reported in other corpora [37,38].

## List of abbreviations used

GO Gene Ontology; IPC International Patent Classification; MAP Mean Average Precision; MeSH Medical Subject Headings; P0 Top-precision; PA Prior Art; TAC Title, Abstract and Claims; TACD Title, Abstract, Claims and Description; TREC Text REtrieval Conferences; TS Technical Survey.

## Competing interests

The authors declare that they have no competing interests.

## Authors' contributions

EP conducted experiments, developed the software components and drafted the manuscript. JG participated in the experiments and helped to draft the manuscript. OK implemented the search engine in the Novartis interface. FO and TV participated in the design and coordination of the project. CL contributed to the choices of onto-terminological resources and helped to draft the manuscript. PR conceived the study, supervised its design and coordination and drafted the manuscript. All authors read and approved the final manuscript.

## Declarations

This work has been supported by Novartis Pharma AG.

This article has been published as part of *BMC Bioinformatics *Volume 15 Supplement 1, 2014: Integrated Bio-Search: Selected Works from the 12th International Workshop on Network Tools and Applications in Biology (NETTAB 2012). The full contents of the supplement are available online at http://www.biomedcentral.com/bmcbioinformatics/supplements/15/S1.
